# Supplementary material for: A Novel Monoclonal Antibody Targeting Cancer-Specific Plectin Has Potent Antitumor Activity in Ovarian Cancer
Source: Cells. 2021 Aug 27;10(9):2218. doi: 10.3390/cells10092218 (PMC8466582; doi:10.3390/cells10092218)
Supplement: Supplementary file 1 [file cells-10-02218-s001.zip › cells-1337259-supplementary.pdf]

cells

Supplemental Figures

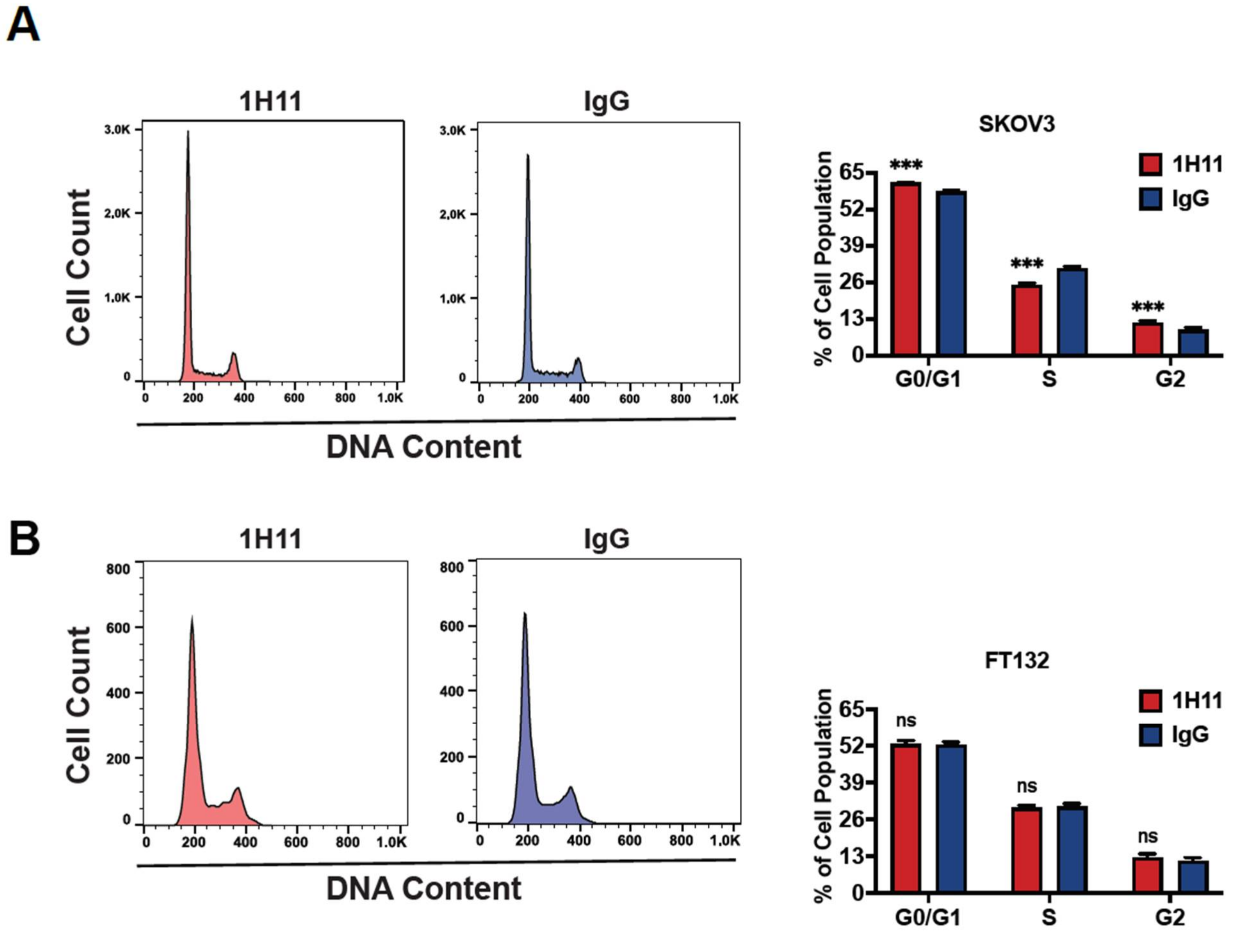

Supplementary Figure S1. 1H11 selectively induces G0/G1 arrest. Cells were treated with 125nM of 1H11 or IgG for 72 hours, then stained with propidium iodide and read out by flow cytometry. 1H11 induced significant G0/G1 arrest in **(A)** CSP-positive SKOV3 cells but not in **(B)** healthy fallopian tube cells, FT132. All data plotted as mean ± SEM. ***p<0.001, non-significant (ns) vs. IgG control.

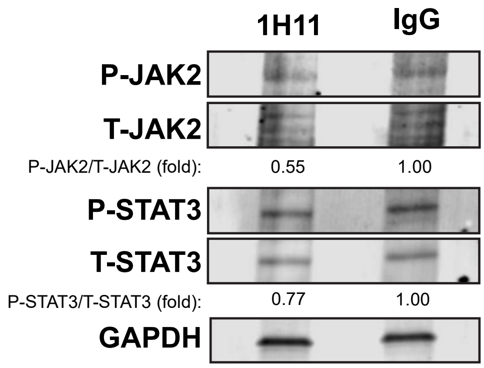

Supplementary Figure S2. 1H11 dampens the JAK2-STAT3 pathway in SKOV3. SKOV3 cells were serum-starved prior incubation with 1H11 or IgG for 10 minutes and subsequent lysis. Western blot analysis demonstrated that 1H11 decreases activation of JAK2 and STAT3.

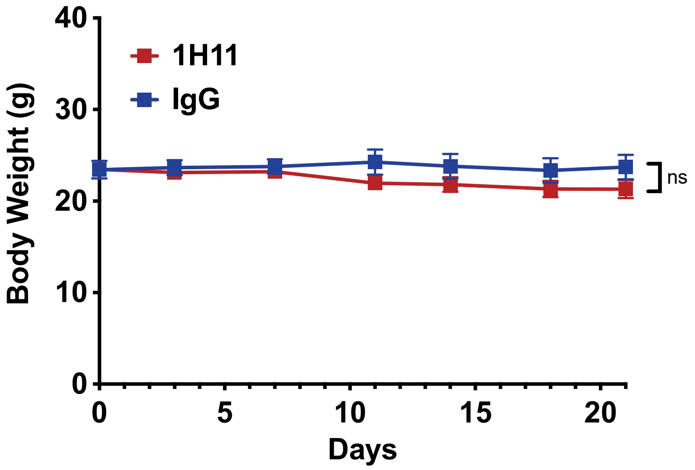

Supplementary Figure S3. Mouse weights of 1H11 and IgG treated mice were not statistically different. The mouse weight of athymic nude mice with OVCAR8 subcutaneous tumors throughout the course of treatment with either 5mg/kg 1H11 or IgG. All data plotted as mean ± SEM, non-significant (ns) vs IgG.

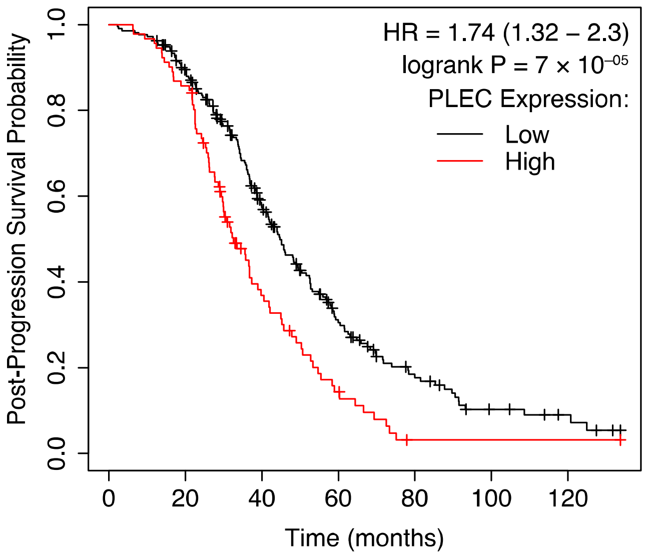

Supplementary Figure S4. Kaplan-Meier plot of the prognostic value of plectin expression on the post-progression survival of patients with serous mutant TP53 OC who previously received platinum-based chemotherapy.

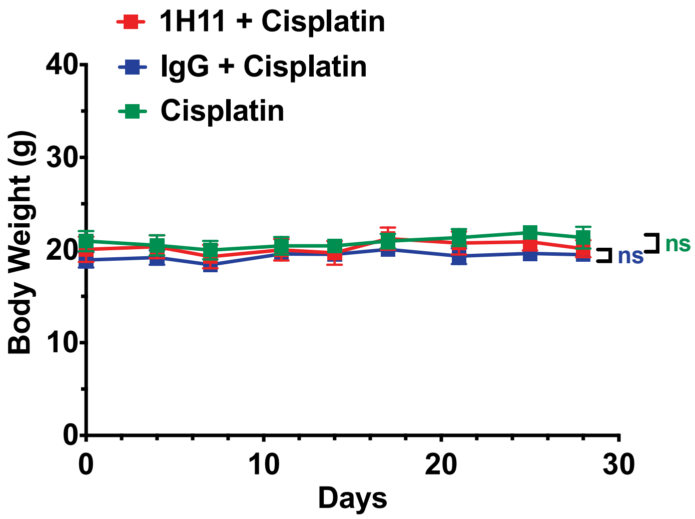

Supplementary Figure S5. Mouse weight was not significantly different between treatment groups. The mouse weight of athymic nude mice with OVCAR8 subcutaneous tumors throughout the course of treatment with either 1) 1mg/kg 1H11 + 2mg/kg cisplatin, 2) 1mg/kg IgG + 2mg/kg cisplatin or 3) 2mg/kg cisplatin alone. All data plotted as mean ± SEM, non-significant (ns) vs. 1H11 + cisplatin.
